# Vitamin D increases remyelination by promoting oligodendrocyte lineage differentiation

**DOI:** 10.1002/brb3.1498

**Published:** 2019-12-13

**Authors:** Ulises Gomez‐Pinedo, Jesús Adriel Cuevas, María Soledad Benito‐Martín, Lidia Moreno‐Jiménez, Noelia Esteban‐Garcia, Laura Torre‐Fuentes, Jordi A. Matías‐Guiu, Vanesa Pytel, Paloma Montero, Jorge Matías‐Guiu

**Affiliations:** ^1^ Neurobiology Laboratory Department of Neurology Institute of Neurosciences IdISSC Hospital Clínico San Carlos Universidad Complutense de Madrid Madrid Spain

**Keywords:** lysolecithin, multiple sclerosis, neurogenesis, oligodendrogenesis, remyelination, vitamin D

## Abstract

**Introduction:**

Several experimental studies have suggested the potential remyelinating effects of vitamin D (VitD) supplements regardless of the presence of VitD deficiency. This study aims to analyze neurogenesis in a model of toxic demyelination in order to evaluate the effects of VitD on demyelination and remyelination.

**Material and methods:**

We used 24 male Wistar rats that had received surgical lesions to the corpus callosum and were injected with lysolecithin. Rats were divided into three groups: Group 1 included eight rats with lesions to the corpus callosum but not lysolecithin injections (sham group), group 2 included eight rats with lesions to the corpus callosum that were injected with lysolecithin (lysolecithin group), and group 3 included eight rats with lesions that were injected with lysolecithin and received VitD (VitD group). We analyzed neurogenesis both in the subventricular zone and at the lesion site.

**Results:**

Administration of VitD promotes the proliferation and differentiation of neural stem cells in the subventricular zone and the migration of these cells to the lesion site in the corpus callosum; these cells subsequently differentiate into oligodendrocyte lineage cells and produce myelin basic protein. This phenomenon was not caused by microglial activation, which was less marked in rats receiving VitD. Megalin expression did not increase at the lesion site, which suggests that VitD is internalized by other mechanisms.

**Conclusion:**

Our results support the hypothesis that regardless of the presence of VitD deficiency, treatment with VitD may contribute to remyelination by promoting the proliferation of oligodendrocyte precursor cells.

## Graphical abstract

Vitamin D promotes remyelination in rats with lysolecithin‐induced demyelination. Vitamin D promotes differentiation into oligodendrocyte lineage cells. Vitamin D promotes proliferation and differentiation of neural stem cells.

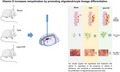

## 1 INTRODUCTION

Remyelination is a target for the treatment of multiple sclerosis. The quest for new strategies for resolving and improving symptoms and protecting axons from further damage represents a new approach (Matías‐Guiu, Oreja‐Guevara, Matías‐Guiu, & Gómez‐Pinedo, 2018; Schultz et al., 2017). Plasma levels of vitamin D (ViD) precursors in patients with MS point to an association between VitD deficiency and MS (Ascherio et al., 2014; Mokry et al., 2015; Pierrot‐Deseilligny & Souberbielle, 2013). According to studies into experimental autoimmune encephalomyelitis (EAE), VitD may play an immunomodulatory role in MS (Adzemovic, Zeitelhofer, Hochmeister, Gustafsson, & Jagodic, 2013; Chiuso‐Minicucci et al., 2015; Lemire & Archer, 1991; Mimura et al., 2016). However, other studies suggest that VitD may also be directly involved in remyelination, by promoting oligodendrocyte progenitor cell (OPC) differentiation (de la Fuente et al., 2015; Matías‐Guiu et al., 2018; Shirazi, Rasouli, Ciric, Rostami, & Zhang, 2015). Several findings support this hypothesis. The VitD receptor (VDR) is expressed in neural stem cells (NSC) (de la Fuente et al., 2015), OPC (Baas et al., 2000; Eyles, Smith, Kinobe, Hewison, & McGrath, 2005), and oligodendrocytes (Eyles et al., 2005), and VDR expression is elevated in MS demyelinating plaques. Likewise, VDR heterodimerises with RXR‐γ in OPC and participates in OPC differentiation (Huang et al., 2011); blocking VDR reduces OPC differentiation in vitro, and administration of VitD promotes NSC proliferation (Gu, Wang, Zhao, & Li, 2015). The purpose of our study was to analyze neurogenesis and oligodendrogenesis in animals with lysolecithin‐induced demyelination (Birgbauer, Rao, & Webb, 2004) evaluating the potential effects on demyelination and remyelination of preventive VitD administration before the injury, with a view to future applications in humans.

## 2 MATERIAL AND METHODS

### 2.1 Animals

We used 24 male Wistar rats (Charles River Laboratories) aged 3–4 months at the beginning of the study. Rats were housed in cages in our hospital's animal facilities with ad libitum access to food (commercial rodent feed) and water, a 12:12 light–dark cycle, and appropriate humidity and temperature.

Three experimental groups were established. Group 1 included eight rats with surgical lesions to the corpus callosum (CC) and no lysolecithin injection (sham group), group 2 included eight rats with surgical lesions that were injected with lysolecithin (lysolecithin group), and group 3 included eight rats with surgical lesions that were injected with lysolecithin and received VitD (VitD group).

### 2.2 Vitamin D administration

Animals in the VitD group received oral VitD supplementation (cholecalciferol): 5,000 IU kg^−1^ day^−1^ of VitD dissolved into 300 µl Elix water, which was previously sonicated and homogenized. VitD was administered for 30 days (15 days prior to lysolecithin injection and 15 days after the procedure) in order to guarantee the presence of VitD at the time of the injury.

### 2.3 Lysolecithin injection

Rats were anesthetised with fentanyl (0.3 mg/kg) plus medetomidine (0.3 mg/kg), and anesthesia was maintained with 2% isoflurane. Rats were then placed in a stereotactic frame. We first determined the position of the bregma, then used stereotactic coordinates to locate the CC. The coordinates used to locate the CC were −0.48 mm anteroposterior, −1.2 mm, and 2.9 mm dorsoventral (Paxinos and Watson). A diamond drill was used to make a 1.5 mm–diameter hole. After drilling, the needle of the syringe was placed at the site indicated by the coordinates and 10 µl of physiological saline solution with 1% lysolecithin (Lysophosphatidylcholine, Sigma L1381) was injected. The wound was then closed with cellulose to achieve hemostasis, and the skin was sutured with single‐layer suturing using 2‐0 silk (interrupted stitches). At the time of surgery, all rats received an intraperitoneal injection of 50 mg/kg body weight of BrdU (Sigma B9285) diluted in saline solution.

### 2.4 Clinical variables

Rats were weighed weekly on a digital scale. Beginning 1‐week postsurgery, motor function was assessed with the inclined plane test, performed every 3 days by a blinded researcher until 1 day before euthanasia. Although this test was initially developed to assess motor function in animal models of spinal cord injury, we deemed it appropriate for our study. Rats were placed horizontally, perpendicular to the major axis of the inclined plane, and we recorded the steepest angle at which rats were able to stay on the inclined plane for 5 s without falling. To evaluate the strength of all four legs equally, we first placed rats in a right‐headed position and then in a left‐headed position. Angles below 70º were considered to represent motor alterations.

### 2.5 Euthanasia

Six weeks after the surgical injury, rats were deeply anesthetised with an intraperitoneal injection of pentobarbital (60 mg/kg), with fentanyl (0.3 mg/kg) administered as an analgesic. Rats were euthanized, and their brains fixed by intracardiac perfusion with 4% paraformaldehyde in 0.1 M PBS. Once fixed, rats' brains were removed, washed 0.1 M PBS, and cryoprotected with 30% sucrose and Tissue‐Tek^®^ OCT compound.

### 2.6 Histological processing

The brain area located between coordinates 1.5 and −1.5 from bregma was sectioned in 40 μm coronal slices; this area exhibited a demyelinating lesion affecting the CC and motor cortex. Brains were sectioned using a cryostat (Microm HM 505E), and sections were placed in a cryopreservation solution for brain tissue containing ethylene glycol and dimethyl sulfoxide.

### 2.7 Immunofluorescence study

Brain sections were washed in PBS, treated with a permeabilisation solution (0.1% Triton X‐100 in 0.1M PBS), and incubated in blocking solution (10% goat serum in PBS) for 30 min at room temperature to prevent nonspecific binding. Slices to be analyzed for BrdU expression were previously immersed in 2N HCl for 20 min. Slices were subsequently incubated overnight at 4ºC with the corresponding primary antibodies. Table S1 lists the antibodies used in the immunohistochemical study. After incubation with primary antibodies, slices were washed three times in PBS to remove excess primary antibodies and subsequently incubated for 2 hr at room temperature using a secondary antibody specific to each primary antibody (mouse, rat, rabbit, or goat), conjugated to a fluorophore, such as Cy3 (1:1,000, Jackson) or Alexa Fluor 488, 555, or 647 (1:1,500, Invitrogen). All antibodies were diluted in PBS. Sections were washed three times in PBS and counterstained with DAPI nuclear stain (1 μg/ml 4′,6‐Diamidino‐2‐phenylindole dihydrochloride; Sigma‐Aldrich) at a dilution of 1:5,000 for 10 min. They were then mounted on gelatin‐coated slides and covered with FluorSave (Calbiochem), a mounting medium for fluorescence. Each marker was used in three slices.

The immunohistochemical study was performed by blinded researchers in all cases, using confocal microscopy (Olympus AF1000); we analyzed 6–8 serial sections per animal. We recorded the number of cells positive for each marker in each section and calculated the means. In experiments with the BrdU marker, positive cells were classified according to location: Cells located in the SVZ and those located in the CC at the lesion site. The number of immunoreactive cells was calculated by counting all positive cells in a randomly selected 1‐mm^2^ area from each section; three different slices were used for each area studied (motor cortex, CC, and SVZ dorsal). All confocal microscopy images were obtained by sequential scanning to minimize emission bleed‐through.

We quantified myelin basic protein (MBP) and proteolipid protein (PLP) by optical density (black/white binary images), determining the percentage of positive cells per field. Eight areas were randomly selected per animal using the ImageJ software, version 1.42 (USA).

### 2.8 Statistical analysis

Immunohistochemical and clinical data were analyzed with ANOVA and Tukey's post hoc test. Statistical analysis was performed with StatView 5.0 and GraphPad Prism software. Graphs show mean values for each group; *SE* are expressed as error bars.

### 2.9 Ethical approval

Animals were manipulated according to the ethical standards of the Spanish Ethics Committee (RD 1201/2005) and in compliance with European Directive 86/609/EEC on the protection of animals used for scientific purposes. Our study was approved by our hospital's Research Committee and Animal Ethics Committee.

## 3 RESULTS

### 3.1 Vitamin D prevents lysolecithin‐induced clinical alterations

At week 6, rats in the lysolecithin group displayed discrete though statistically significant weight loss compared with the other two groups. At week 3, this group displayed statistically significant differences in inclined plane test performance, compared with the group receiving VitD, which showed no significant differences compared with the sham group (Figure S1).

### 3.2 Vitamin D promotes cell proliferation in the SVZ during demyelination

Rats in the VitD group (group 3) exhibited more BrdU+ cells than the other two groups (Figure S2). Group 1 displayed a mean (*SE*) of 81.2 (2.4) BrdU+ cells, group 2, 92.0 (3.5) cells, and group 3, 103.9 (2.9) cells; differences between groups were statistically significant (Figure S2).

**Figure 1:**
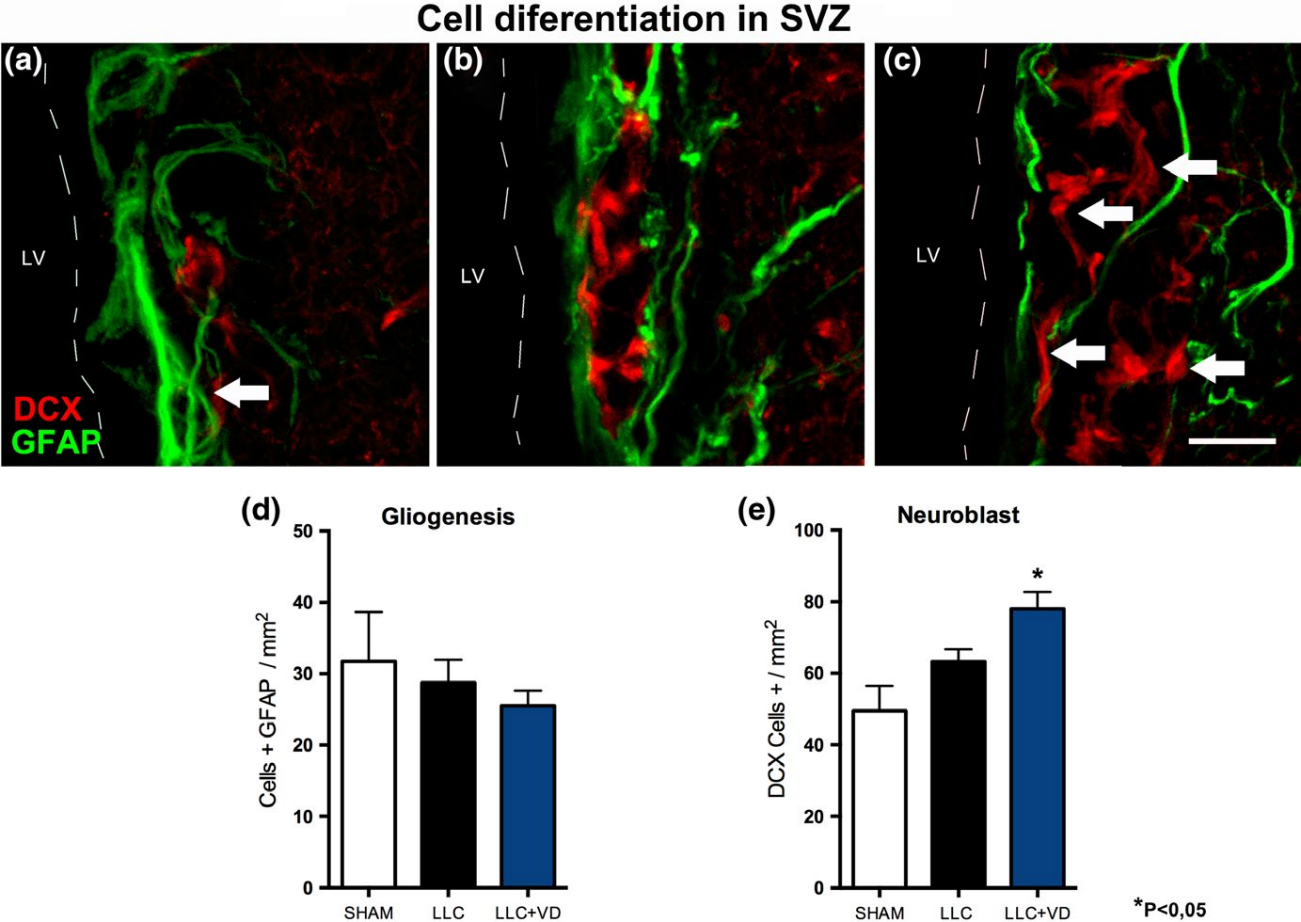
Cell differentiation in the SVZ. (a–c) Immunohistochemical study; confocal microscopy images of cell differentiation in the SVZ in the sham (a), lysolecithin (b), and vitamin D groups (c). The sham group displayed increased GFAP expression and chains of DCX+ cells parallel to the ventricular lumen (arrow). In groups 2 and 3, lysolecithin increased the expression of DCX+ cells; the image shows chains of DCX+ cells surrounded by GFAP cells. Animals in group 3 displayed increased expression of DCX+ cells in the SVZ; the images show up to 3 or 4 layers of cells (arrows). Astrocytes appear surrounding the chains of DCX+ migrating cells. (d–e) Quantification of GFAP+ (d) and DCX+ cells (e). Although the number of GFAP+ cells in the SVZ increased slightly in the sham group and was smaller in the vitamin D group, differences between the groups were not statistically significant (d). The group receiving vitamin D displayed a statistically significant increase in the number of neuroblasts (DCX+) compared with the other 2 groups (e). Rats in the lyslecithin group displayed larger numbers of neuroblasts than rats in the sham group; however, differences were not statistically significant. Data are expressed as means (*SE*). *p < .05. Scale bar = 50 µm. Line dot: ventricular wall

In the analysis of cell differentiation in the SVZ, cells simultaneously expressing BrdU and GFAP did not display increased GFAP expression in any group (group 1:31.7 [6.8]; group 2:28.7 [3.2]; and group 3:25.5 [2.1]), which suggests that astrocyte lineage differentiation was not increased. In contrast, group 3 showed significantly more BrdU+/DCX+ cells along the ventricular wall (group 1:49.5 [6.9]; group 2:63.2 [3.2]; group 3:78.0 [4.7]), as shown in Figure 1.

### 3.3 Vitamin D promotes oligodendrocyte differentiation and neuroblast migration to the lesion site and reduces demyelination

**Figure 2:**
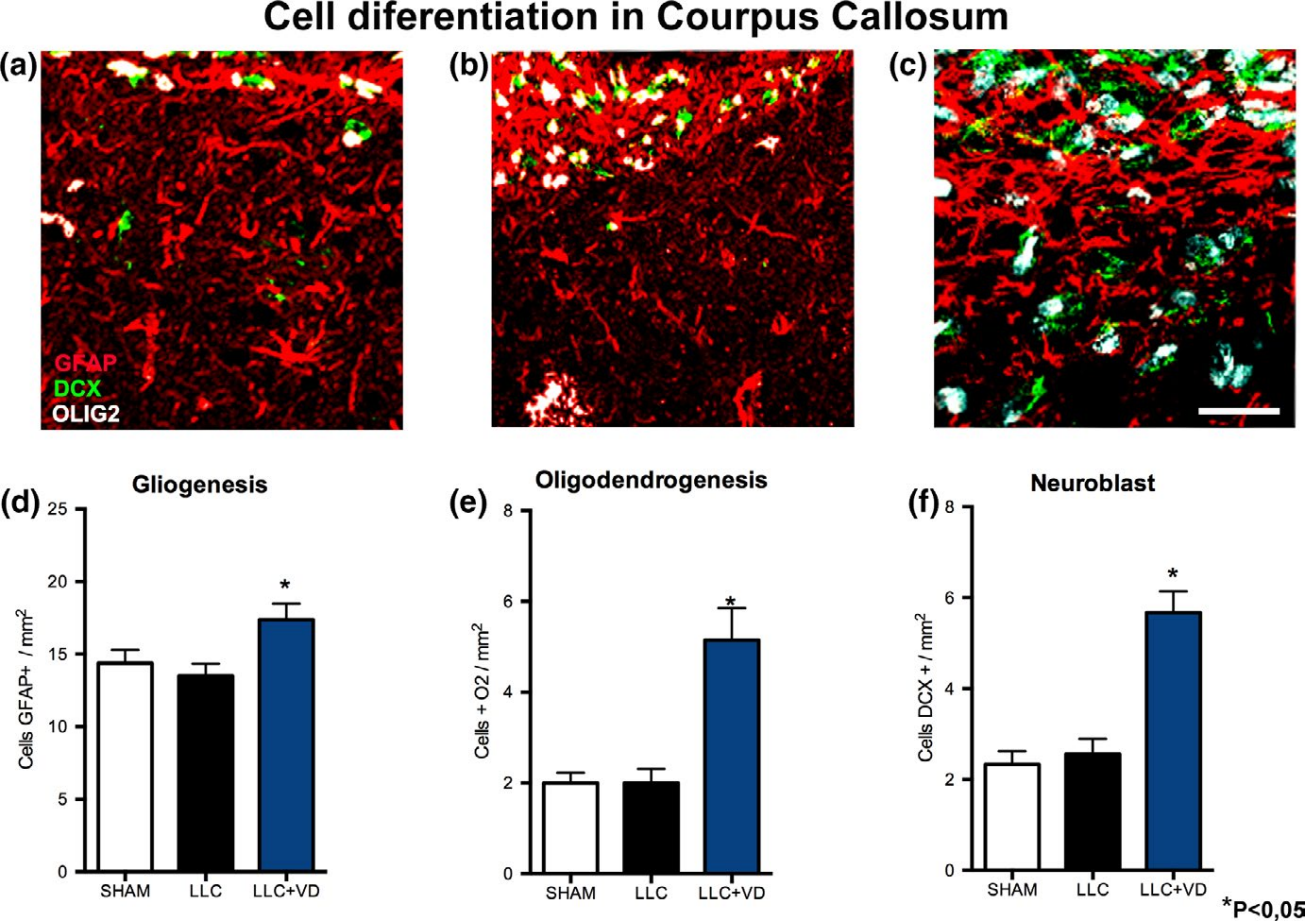
Cell differentiation at the lesion site. The group receiving vitamin D showed a larger number of GFAP+, Olig2+, and DCX+ cells at the lesion site (a–c); these differences were statistically significant. Analysis of images of the corpus callosum (d–f) shows that DCX+ cells in group 3 were fusiform and colonized the lesion site. We also observed an increase in the number of GFAP+ cells, but decreased GFAP expression. In groups 1 and 2, GFAP+ cells formed a scar and exhibited increased expression of cytoskeletal proteins. DCX+ cells in groups 1 and 2 were less fusiform and had fewer processes. The number of Olig2+ cells was larger in the group receiving vitamin D. Data are expressed as means (*SE*). *p < .05. Scale bar = 50 µm

The CC of rats in group 3 displayed significantly larger numbers of BrdU+ cells than the other two groups (mean [*SE*]; group 1:16.7 [1.1]; group 2:16.1 [1.0]; and group 3:21.0 [0.9]) (Figure S3). Rats in group 3 also displayed slightly larger numbers of BrdU+/GFAP cells (group 1:14.3 [0.9]; group 2:13.5 [0.8]; and group 3:17.0 [1.0]); differences between groups were statistically significant. However, these rats also displayed larger numbers of neuroblasts (DCX+), suggesting that these cells migrated to the lesion site (group 1:2.3 [0.2]; group 2:2.5 [0.3]; and group 3:5.6 [0.4]). Likewise, group 3 displayed more Olig2+ cells, indicating that these cells had differentiated into oligodendrocyte lineage cells (group 1:2.0 [0.2]; group 2:2.0 [0.8]; and group 3:5.0 [0.7]) (Figure 2). The significant decrease in the number of neurons (NeuN+) undergoing apoptosis (Cas3+) in group 3 compared with group 2 (group 1:17.2 [1.8]; group 2:33.5 [5.1]; and group 3:24.5 [1.5]) also indicates oligodendrocyte differentiation (Figure S4).

**Figure 3:**
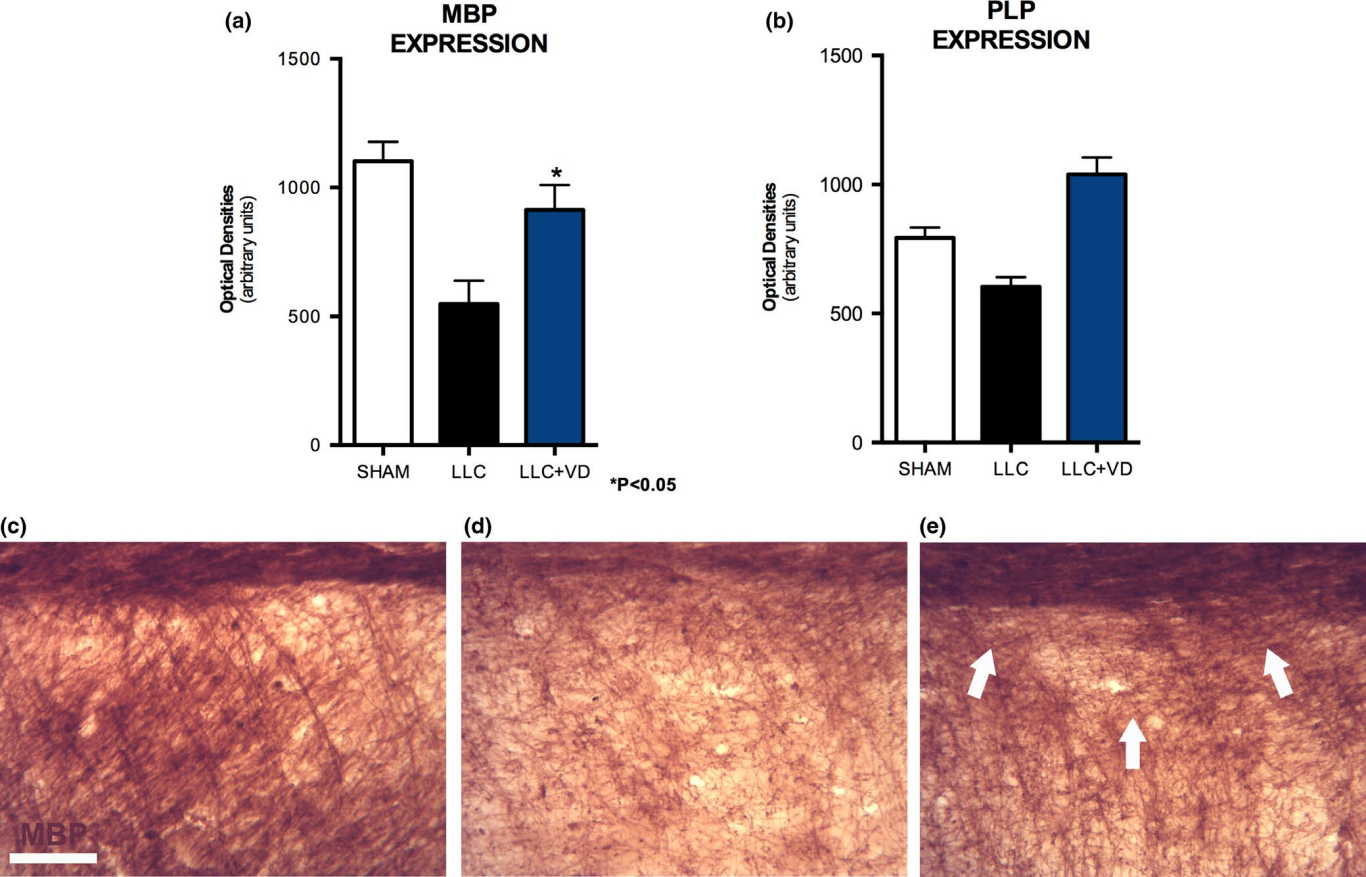
MBP and PLP expression at the lesion site in the 3 experimental groups. Rats in group 3 displayed increased MBP expression (increased density of bundles of myelinated axons in the corpus callosum and in layer VI adjacent to the lesion site (arrows in e), showing similar organization to healthy tissue (c), with compact neuropil and with sheaths organized in a column. The opposite was observed in group 2 (d), which displayed wide spaces in the parenchyma and dispersed MBP+ fibers; the neuropil was edematous and lax, not compact. These differences were statistically significant (a: MBP, b: PLP), suggesting that vitamin D supplementation reduces demyelination. Data are expressed as means (*SE*). *p < .05. Scale bar = 250 µm

Rats in group 2 exhibited lower MBP expression (548.2 [90.58] OD) at the lesion site, as described in the model. Differences in MBP expression between groups 1 (1,103.0 [74.91] OD) and 2 were statistically significant. Furthermore, MBP expression was significantly higher in group 3 (913.4 [96.9] OD) than in group 2 (Figure 3), which suggests that VitD reduces myelin loss.

Animals injected with lysolecithin showed lower PLP expression (603.8 [36.68] OD) at the lesion site. A significant difference was observed between groups 1 and 2, with the sham group showing reduced PLP expression compared with the VitD group (843.8 [40.29] OD) and VitD animals showing better results (1,040 [65.05] OD) (Figure 3), which confirms that VitD reduces myelin loss and promotes remyelination.

### 3.4 Vitamin D increases the number of oligodendrocyte progenitor cells at the lesion site

NG2 expression at the lesion site was significantly different in animals from groups 1 and 2 (mean [*SE*]: 22 [2.3] vs. 27.5 [2.2] cells). Animals in group 3 showed a significantly higher density of cells expressing NG2 (33 [3]) (Figure S5); these cells colocalized with GFAP‐positive cells.

### 3.5 Vitamin D supplementation decreases lysolecithin‐induced Iba1+ cell expression

Rats in group 2 showed a statistically significant increase in the expression of Iba1+ cells compared with those in group 1 (group 1:28.0 [2.8] and group 2:34.7 [1.9]), displaying amoeboid characteristics. The expression of Iba1+ cells was reduced with VitD supplementation: It was less marked in group 3 than in the sham group (22.0 [1.6]), showing an inactive, ramified phenotype (Figure S6).

### 3.6 Vitamin D supplementation does not increase LRP2 expression

The expression of megalin receptors (LRP2) was similar across groups and in all marked cells (GFAP+, DCX+, Olig2+, NeuN+) (Figure S7).

## 4 DISCUSSION

Our study analyses the impact of VitD supplementation on neurogenesis in the SVZ and at the lesion site in an animal model of lysolecithin‐induced demyelination with a view to understanding the mechanisms underlying demyelination in MS. Multiple studies have analyzed the effects of VitD on experimental models of central and peripheral nervous system demyelination (Chabas et al., 2013; Goudarzvand, Javan, Mirnajafi‐Zadeh, Mozafari, & Tiraihi, 2010; Mashayekhi & Salehi, 2016; Montava et al., 2015; Nystad, Torkildsen, & Wergeland, 2018; Nystad et al., 2014; Wergeland et al., 2011) (Table 1). Our study is the first to use a model of lysolecithin‐induced demyelination; furthermore, we used male rats, which challenges the hypothesis that VitD confers protection to female animals only (Spach & Hayes, 2005). The results of these studies contradict the idea that the effectiveness of VitD supplements depends on the presence of VitD deficiency (Groves, McGrath, & Burne, 2014). However, it may be hypothesized that VitD has a beneficial impact on myelination independently of any existing deficiency, given that the previously mentioned studies used animal models with normal VitD levels. VitD deficiency has been failed to alter neurogenesis (Groves et al., 2016; Groves & Burne, 2017), which raises the question of whether VitD supplementation may promote neurogenesis. A study by Nystad (Nystad et al., 2018) reports a favorable effect in a cuprizone model. VitD was found to promote myelin repair in a model of cuprizone‐induced demyelination, probably due to the hormone's ability to promote oligodendrocyte differentiation.

**Table 1:** Studies analyzing the effects of VD supplementation in animal models of demyelination

Author/year	Model/CNS or PNS	Animal/sex/no. animals per group	VD dose/administration	Conclusions
Chabas et al. (2013)	Surgical lesion to a peripheral nerve (peroneal nerve)/PNS	Sprague Dawley rats/male/6 (6 additional rats in the group receiving 500 IU kg^−1^ day^−1^ cholecalciferol in the final stage)	Ergocalciferol dosed at 100 IU kg^−1^ day^−1^ or 500 IU kg^−1^ day^−1^, or cholecalciferol dosed at 100 IU kg^−1^ day^−1^ or 500 IU kg^−1^ day^−1^	Increased remyelination at the site of the lesion
Montava et al. (2015)	Surgical lesion to a peripheral nerve (facial nerve)/PNS	New Zealand white rabbits/male/8	200 IU kg^−1^ day^−1^ for one week/oral route	Increased myelination
Goudarzvand et al. (2010)	Ethidium bromide/CNS	Sprague‐Dawley rats/male/not specified	Intracranial injection of 5 μg/kg VD3 at days 2, 7, and 28 after the lesion	Increased remyelination of the hippocampus
Wergeland et al. (2011)	Cuprizone/CNS	C57Bl/6 mice/female/18	(a) < 76 IU/day; (b) 760 IU/day; (c) 9,425 IU/day; (d) 19,003 IU/day/oral route	High doses of VD reduced demyelination in the corpus callosum and attenuated microglial activation.
Nystad et al. (2014)	Cuprizone/CNS	C57Bl/6 mice/female/18	0.2 µg calcitriol/intraperitoneal injections twice a week for 3 weeks	Remyelination regardless of immune effects
Mashayekhi and Salehi (2016)	Cuprizone/CNS	Balb/c mice/female/11	5 µg/kg VD3 for 6 weeks/intraperitoneal injection	Increased CNPase and MOG expression
Nystad et al. (2018)	Cuprizone/CNS	Five‐week‐old female c57Bl/6 mice, *n* = 72	2 μg calcitriol for 3 weeks/intraperitoneal injection	protective effect on axonal loss and Increased remyelination
Present study (2018)	Lysolecithin/CNS	Wistar rats/male/8	5,000 IU kg^−1^ day^−1^ for 30 days (15 days before lysolecithin injection and 15 days afterward)/oral route	Increased remyelination Increased numbers of oligodendrocyte lineage cells and Olig2+ cells, increased MBP expression

Shirazi et al. (2015) recently reported a similar effect in in vitro models of neurospheres. In addition, in a later study they observed the same effect in a mouse model of EAE (Shirazi et al., 2017). Our results suggest that VitD supplementation promotes NSC proliferation and differentiation into neuroblasts in the SVZ and stimulates neuroblast migration to the lesion site in the CC; these effects have previously been observed in other models, with neuroblasts migrating to the lesion site (Gómez‐Pinedo et al., 2018; Snigdha, Smith, Prieto, & Cotwan, 2012). Newly generated OPCs in the SVZ migrate toward the lesion site in the CC, similarly to the findings of other researchers (Maire, Wegener, Kerninon, & Nait, 2010; Yamashita et al., 2006). These cells would subsequently differentiate into oligodendrocyte lineage cells and generate MBP (Shirazi et al., 2015). Our findings therefore confirm the results reported by Nystad et al. (2018) suggest that VitD supplementation may be used as remyelination therapy. In our study, VitD supplementation led to an increase in the number of astrocytes (GFAP+) in the SVZ and promoted differentiation into neuronal lineage cells (DCX+) and OPCs (Olig2+) at the lesion site; this was not observed in the rats not receiving VitD. The increase in neuroblasts and OPC and the decrease in GFAP expression have previously been reported by other researchers, in in vitro and in vivo models, which supports our hypothesis of the beneficial effects of VitD (Maire et al., 2010; Maki, Liang, Miyamoto, Lo, & Arai, 2013; Yamashita et al., 2006). Animals not treated with VitD displayed increased Cas3 expression in NeuN+ cells, which suggests more pronounced neuronal apoptosis (Snigdha et al., 2012). Several authors consider VitD to be a neuroactive steroid (neurosteroid), involved in processes of regeneration, proliferation, and myelination. Therefore, in our study, VitD plays an important role in oligodendrogenesis/remyelination and in neuroprotection. Neuroprotection mediated by VitD includes the reduction of calcium toxicity and nitric oxide synthesis, modulation of cytokine release, modulation of glutathione, neurotrophin induction, and neuritogenesis, as well as the protection of neurons against cell death. VitD base supplementation may be an effective neuroprotective therapy (Charalampopoulos, Remboutsika, Margioris, & Gravanis, 2008; Garcion, Wion‐Barbot, Montero‐Menei, Berger, & Wion, 2002; Kalueff, Tuohimaa, & Allan, 2007; McGrath, Feron, Eyles, & Mackay‐Sim, 2001). According to some researchers (Kotter, Li, Zhao, & Franklin, 2006), VitD increases microglial activation, which may stimulate the removal of myelin debris and consequently promote remyelination, since myelin inhibits OPC maturation in vitro (Robinson & Miller, 1999). In contrast, our study found lower Iba1+ cell expression in the rats not receiving VitD, which supports the hypothesis that the remyelinating power of VitD depends on increased OPC differentiation.

VD is obtained through intestinal or skin absorption and is converted into 25‐hydroxyvitamin D in the liver and then into 1,25‐dihydroxyvitamin D. Both compounds are transported primarily by the binding of vitamin D. Cell internalization is mediated primarily by the DBP‐megalin complex (LRP2) and is subsequently converted to 25‐hydroxyvitamin D3 by vitamin D 25‐hydroxylase. 1α‐hydroxylase converts this to the active form, and 1,25‐dihydroxyvitamin D3 can convert into the inactive form. 1,25 [OH] 2D3 binds to the nuclear vitamin D receptor (VDR), interacting with the alpha retinoid receptor X (RXRA) to form a heterodimer, allowing transport to theell nucleus (Pytel et al., 2019). Megalin is expressed in neurons and astrocytes (Bento‐Abreu et al., 2008). However, in immune cells, VitD internalization is not mediated by megalin (DeLuca, 2004). Likewise, megalin has been found to be expressed in oligodendrocytes (Wicher et al., 2006) and involved in OPC migration during development (Ortega et al., 2012). We did not detect increased megalin expression in any of the cells; we may hypothesize that VitD acts on these cells through some other mechanism, which may explain the decreased megalin mRNA expression in active and chronic MS lesions compared with normal‐appearing white matter (Smolders et al., 2013).

Now that a wide range of immunomodulatory treatments has been developed, remyelination has become the main therapeutic challenge in MS (Matías‐Guiu et al., 2018). According to our results, treatment with VitD, regardless of the presence of VitD deficiency, may contribute to remyelination by promoting the creation of myelin‐producing oligodendrocyte precursor cells. Our study has several limitations. First, we have not included a group without intervention, although other studies do not include it (Goudarzvand et al., 2010; Nystad et al., 2014; Wergeland et al., 2011). The second limitation refers to the fact that the model of lysolecithin‐induced demyelination, like other demyelination models, has the limitation of being toxin‐induced (Torre et al., 2017) and noninflammatory origin, and although the repair of myelin depends on the activation status of macrophages and microglia. Indeed, in the cuprizone model, microglial cells increase in number and are activated after cuprizone treatment (Goldberg, Clarner, Beyer, & Kipp, 2015), so that is similar to what happens in the MS, and has also been observed in the experimental allergic encephalomyelitis (Lemire & Archer, 1991). VitD may therefore have different effects in inflammatory diseases, such as MS, but it is not possible to evaluate the potential influence of the inflammatory response on the effect of VD. However, we suggest that VitD supplementation may be an effective treatment in patients with diseases causing alterations in oligodendrocyte differentiation, such as Alexander disease, as reported in a recent study by our research group (Gómez‐Pinedo, Sirerol‐Piquer, Duran‐Moreno, Garcia‐Verdugo, & Matías‐Guiu, 2017). Future studies should therefore analyze the effects of VitD supplementation and determine the most effective dose in the context of MS. Our results represent a basis for a future clinical trial with high doses of VitD for remyelination therapy of demyelinating diseases.

## CONFLICT OF INTEREST

None declared.

## AUTHOR CONTRIBUTIONS

UGP and JMG contributed to study design; MSBM, LMJ, JAC, LTF, and NEG contributed to laboratory analysis; JAC and UGP contributed to microscopy and molecular study; UGP and JAMG contributed to statistical analysis; all authors involved in analysis of results and manuscript revision and approval; JAC and UGP designed the figures; UGP, JMG, and JAMG involved in manuscript preparation.

## Data Availability

The data that support the findings of this study are available on request from the corresponding author. The data are not publicly available due to privacy or ethical restrictions.
