# Supplementary material for: Vitamin D increases remyelination by promoting oligodendrocyte lineage differentiation
Source: Brain Behav. 2019 Dec 13;10(1):e01498. doi: 10.1002/brb3.1498 (PMC6955836; doi:10.1002/brb3.1498)
Supplement: Supplementary file 1 [file BRB3-10-e01498-s001.docx]

**Supplementary Material**

**Supplementary Material Table S1.** Antibodies used in the immunohistochemical study.

| **Antibody** | **Dilution / Company / Ref.** | **Description** |
| --- | --- | --- |
| Anti-Iba1 | 1:1000 / Wako / 019-19741 | Calcium-binding protein specifically expressed in microglial cells and upregulated during activation of these cells |
| Anti-GFAP | 1:600 / Millipore / MAB360 | Glial fibrillary acidic protein; intermediate filament protein in astrocytes. GFAP expression increases with astrocyte activation. |
| Anti-Caspase-3 | 1:200 / Millipore / 04-1090 | Apoptosis-inducing caspase common to extrinsic and intrinsic apoptosis pathways |
| Anti-DCX | 1:200 / Santa Cruz / SC-8066 | Neuroblast marker |
| Anti-MBP | 1:500 / Abcam / ab65988 | Myelin basic protein |
| Anti-PLP | 1:200 / Abcam / ab183493 | Phospo Lipid Protein, |
| Anti- NG2 | 1:100 / Abcam / ab50009 | Proteoglycan express in oligodendrocytes progenitors |
| Anti-Olig2 | 1:300 / Abcam / ab81093 | Oligodendrocyte precursor cell marker |
| Anti-BrdU | 1:200 / Abcam / ab6326 | Thymidine analogue and proliferation marker |
| Anti-LRP2 | 1:200 / Abcam / 184676 | Megalin antibody |
| Anti-NeuN | 1:500 / Millipore / MAB377 | Neuronal marker |

**Supplementary Material Figure S1.**


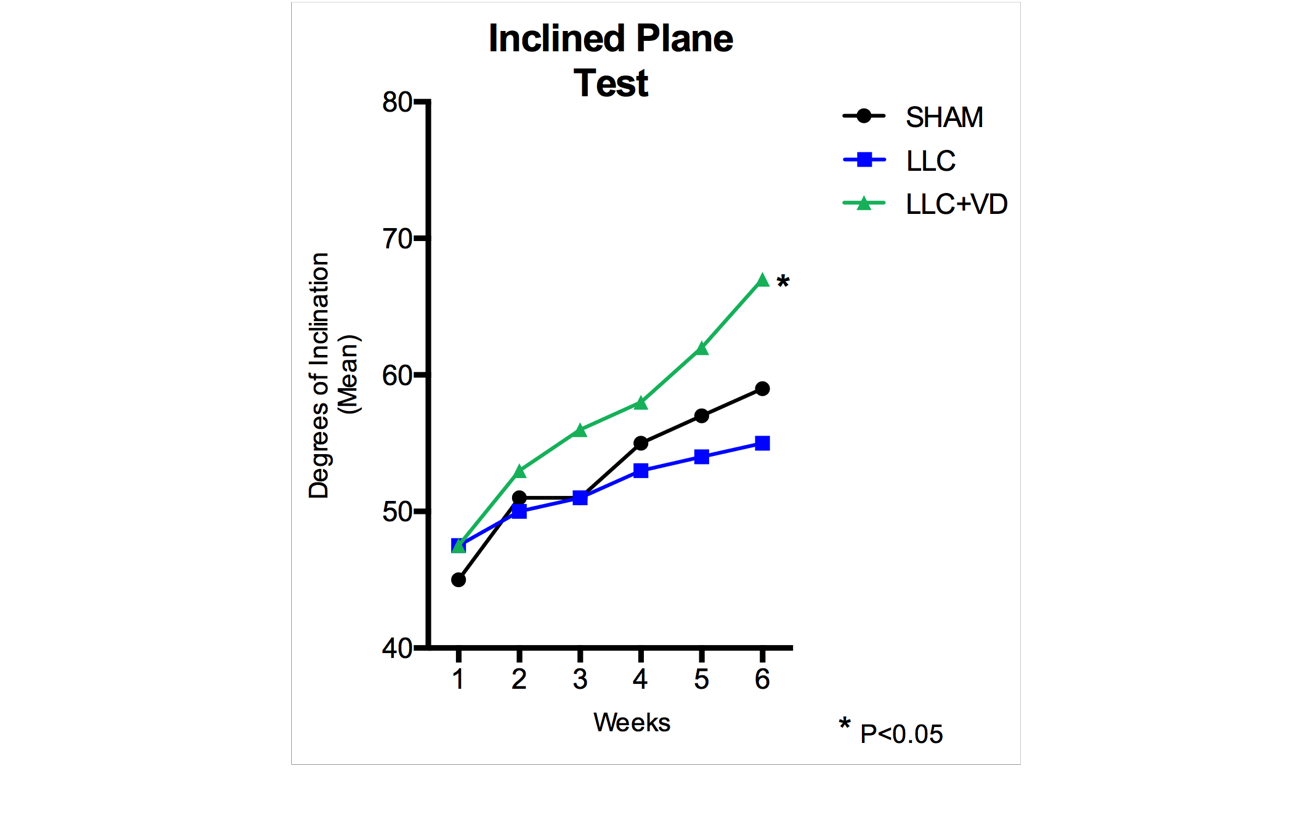


Inclined plane test. At week 3, rats receiving VitD performed similarly to those of the sham group. Differences between rats in groups 2 and 3 were statistically significant (*P< 0.05). Rats in group 3 were able to maintain a stable position up to an angle of 63º until week 6, when they reached an angle of 70º.

**Supplementary Material Figure S2.**

**
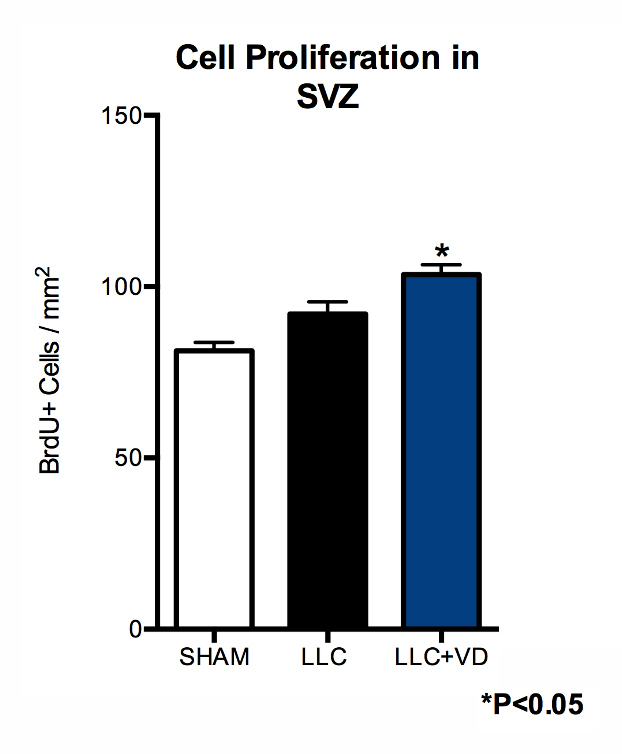
**

Cell proliferation in the SVZ Rats in group 3 displayed a larger number of BrdU+ cells than rats in group 1: the difference was statistically significant. Data are expressed as means ± SE. *P< 0.05.

**Supplementary Material Figure S3.**

**
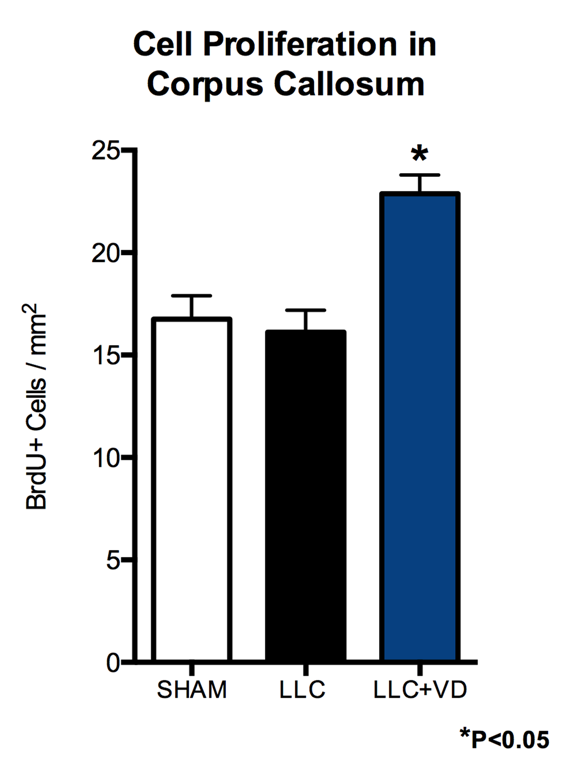
**

Cell proliferation in the corpus callosum. Rats receiving VD displayed significantly more BrdU+ cells in the corpus callosum. Data are expressed as means ± SE. *P < 0.05.

**Supplementary Material Figure S4.**

**

**

Expression of cells colocalising NeuN and Cas3 at the site of the lesion. After lysolecithin injection (groups 2 and 3), rats treated with VD displayed smaller numbers of NeuN+/Cas3+ cells than those in group 2. Furthermore, NeuN+/Cas3+ cells were more abundant along the edges of the demyelinating lesion. Groups 1 and 2 displayed differences in the number of NeuN+/Cas3+ cells, which may suggest that VD supplementation protects neurons close to the lysolecithin-induced lesion against cell death. Data are expressed as means ± SE. *P< 0.05. **P<0.01

**Supplementary Material Figure S5.**

**

**

OPC in the corpus callosum. Rats receiving VD displayed significantly more NG2+ cells in the corpus callosum. Data are expressed as means ± SE. *P< 0.05.

**Supplementary Material Figure S6.**

**
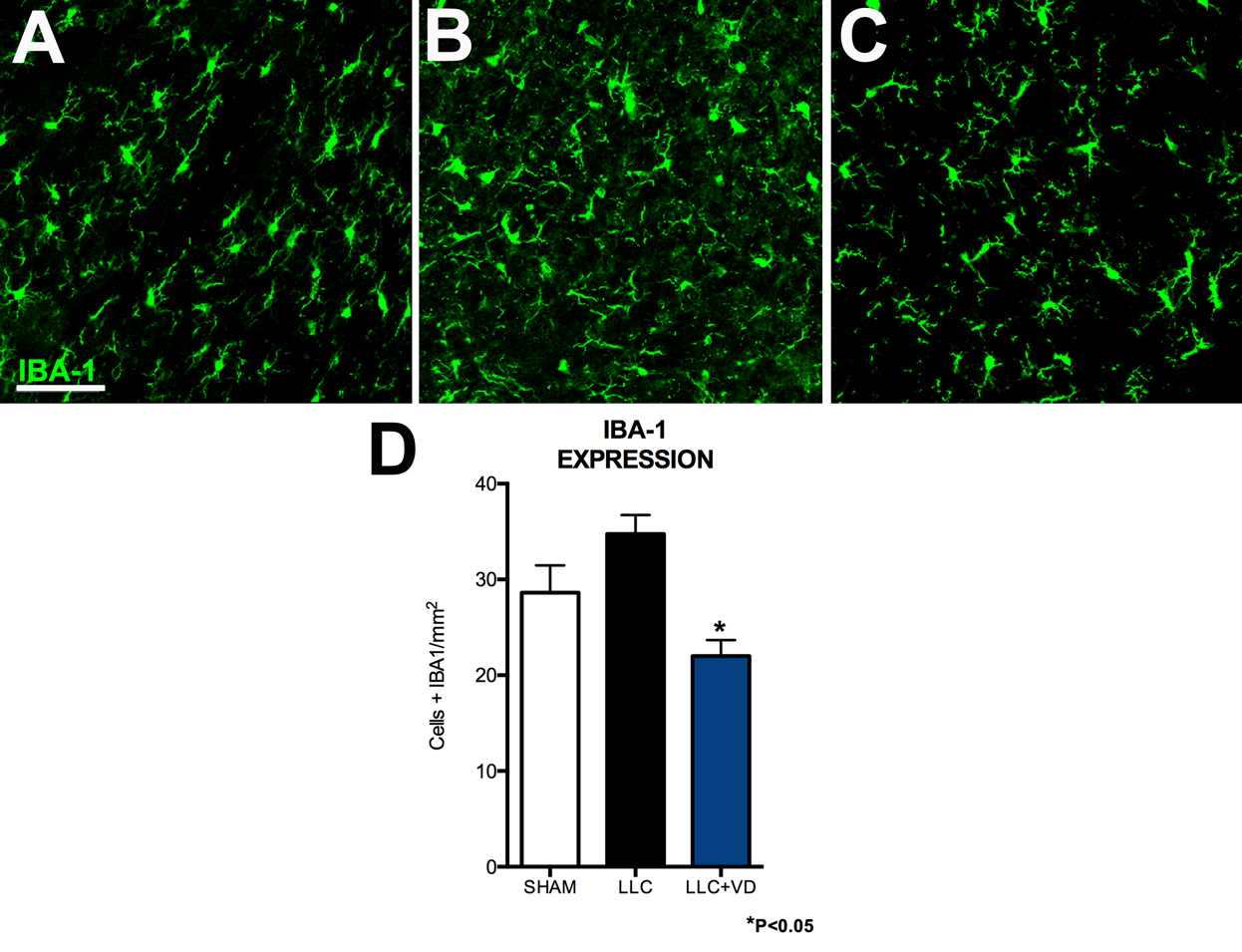
**

Iba1 expression. Confocal microscopy photomicrographs used for the quantitative analysis of the number of microglial cells (A-C). Rats in group 2 displayed increased numbers of cells with an amoeboid and phagocytic phenotype. Cells in groups 1 and 3, in contrast, displayed an arborised, rod-shaped morphology, which suggests that these cells are at a stage of less marked phagocytic activity. Quantitative data show statistically significant differences in the number of microglial cells (Iba1+) along the edge of the lesion (D). Data are expressed as means ± SE. *P< 0.05. Scale bar = 50 µm.

**Supplementary Material Figure S7.**

**
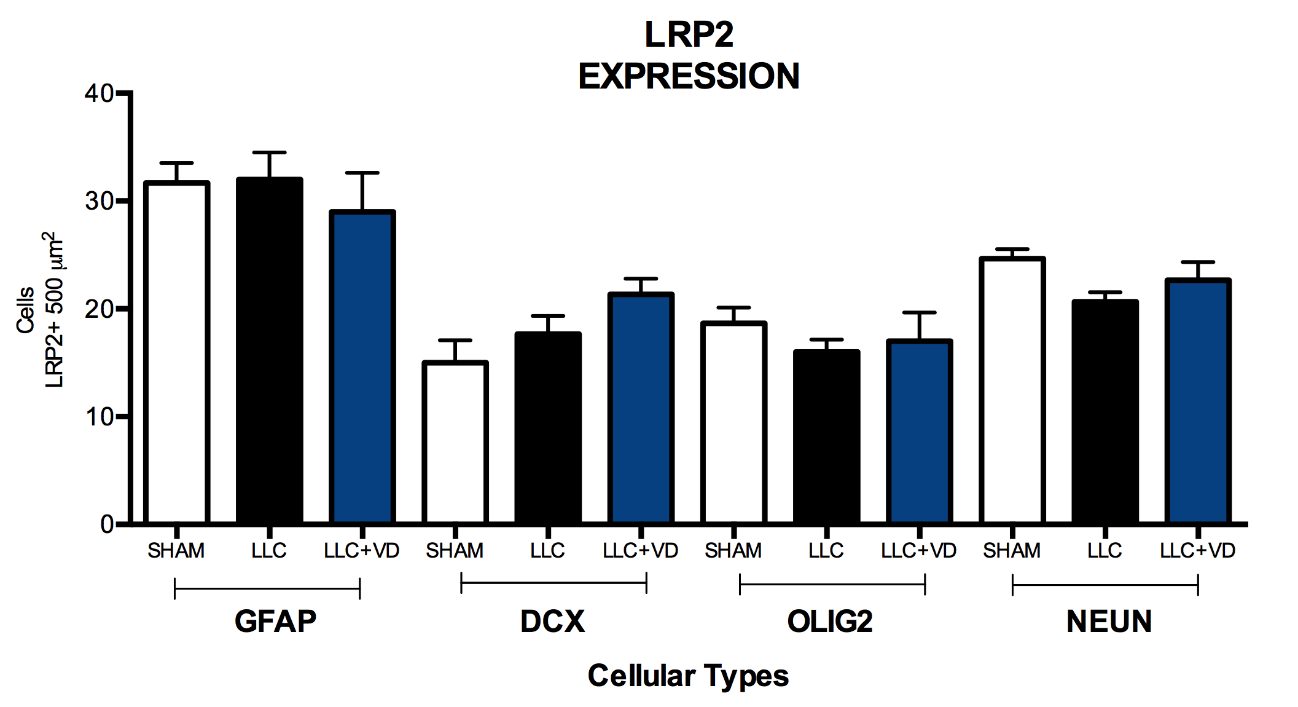
**

Coexpression of megalin (LRP2) at the site of the lesion in each cell type. No differences in the number of cells coexpressing LRP2/GFAP, LRP2/DCX, and LRP2/Olig2 were found between groups for any of the cell types found at the site of the lesion. This may be due to the fact that megalin is not a direct target of VD, since this receptor may have several routes of action. Data are expressed as means ± SE.
